# Computer Vision and Machine Learning for Intelligent Sensing Systems

**DOI:** 10.3390/s23094214

**Published:** 2023-04-23

**Authors:** Jing Tian

**Affiliations:** Institute of Systems Science, National University of Singapore, Singapore 119615, Singapore; tianjing@nus.edu.sg

Intelligent sensing systems have been fueled to make sense of visual sensory data to handle complex and difficult real-world sense-making challenges due to the rapid growth of computer vision and machine learning technologies. We can now interpret visual sensory data more effectively thanks to recent developments in machine learning algorithms. This means that in related research, significant attention is now being paid to problems in this field, such as visual surveillance, smart cities, etc.

The Special Issue offers a selection of high-quality research articles that tackle the major difficulties in computer vision and machine learning for intelligent sensing systems from both a theoretical and practical standpoint. It includes intelligent sensing techniques [1,2,3,4,5], twelve foundational investigations into sense-making methods [6,7,8,9,10], and particular uses of intelligent sensing systems in autonomous driving [11] and virtual reality [12].

**Intelligent sensing techniques**

- Kokhanovskiy et al. [1] demonstrated the application of deep neural networks to process the reflectance spectrum from a fiberoptic temperature sensor.
- Shiba et al. [2] proposed collapse metrics by using the first principles of space–time deformation based on differential geometry and physics for contrast maximization, which provided state-of-the-art results on several event-based computer vision tasks.
- Chen et al. [3] presented a system that integrates mobile edge computing technology and simultaneous wireless information and power transfer technology to improve the service supply capability of WSN-assisted IoT applications.
- Niu et al. [4] proposed a new fusion network to minimize the influences from the two most common sensor noises, i.e., depth noises and pose noises.
- Hashmani et al. [5] presented a novel SLIC extension based on a hybrid distance measure to retain content-aware information for semi-dark images.

**Intelligent sense-making techniques**

- Le and Scherer [6] performed a comprehensive survey of many studies, methods, datasets, and results for human segmentation and tracking in video.
- Tran et al. [7] proposed a heuristic attention representation learning framework relying on the joint embedding architecture, in which the two neural networks are trained to produce similar embedding results for different augmented views of the same image.
- Zaferani et al. [8] proposed a method for the automatic hyper-parameter tuning of a stacked asymmetric auto-encoder to extract personality perception from speech.
- Hu et al. [9] developed two attention modules that work together to extract the coordination characteristics in the process of motion and strengthen the attention of the model to the more important joints in the process of moving.
- Oh et al. [10] suggested estimating gaze by detecting eye region landmarks through a single eye image. It learns representations of images at various resolutions, and the self-attention module is used to obtain a refined feature map with better spatial information.

**Applications of intelligent sensing systems**

- Song and Lee [11] studied autonomous driving and proposed a novel algorithm for online self-calibration between sensors using voxels and three-dimensional convolution kernels.
- Moreno-Armendáriz et al. [12] described a system composed of deep neural networks that analyze characteristics of customers based on their face, as well as the ambient temperature, to generate a personalized signal to potential buyers who pass in front of a beverage establishment.

In conclusion, through the wide range of research presented in this Special Issue, we would like to boost fundamental and practical research on applying computer vision and machine learning for intelligent sensing systems.
